# Stimulus Roving and Flankers Affect Perceptual Learning of Contrast Discrimination in *Macaca mulatta*

**DOI:** 10.1371/journal.pone.0109604

**Published:** 2014-10-23

**Authors:** Xing Chen, Mehdi Sanayei, Alexander Thiele

**Affiliations:** Institute of Neuroscience, Newcastle University, Newcastle-upon-Tyne, United Kingdom; University of Verona, Italy

## Abstract

‘Stimulus roving’ refers to a paradigm in which the properties of the stimuli to be discriminated vary from trial to trial, rather than being kept constant throughout a block of trials. Rhesus monkeys have previously been shown to improve their contrast discrimination performance on a non-roving task, in which they had to report the contrast of a test stimulus relative to that of a fixed-contrast sample stimulus. Human psychophysics studies indicate that roving stimuli yield little or no perceptual learning. Here, we investigate how stimulus roving influences perceptual learning in macaque monkeys and how the addition of flankers alters performance under roving conditions. Animals were initially trained on a contrast discrimination task under non-roving conditions until their performance levels stabilized. The introduction of roving contrast conditions resulted in a pronounced drop in performance, which suggested that subjects initially failed to heed the sample contrast and performed the task using an internal memory reference. With training, significant improvements occurred, demonstrating that learning is possible under roving conditions. To investigate the notion of flanker-induced perceptual learning, flanker stimuli (30% fixed-contrast iso-oriented collinear gratings) were presented jointly with central (roving) stimuli. Presentation of flanker stimuli yielded substantial performance improvements in one subject, but deteriorations in the other. Finally, after the removal of flankers, performance levels returned to their pre-flanker state in both subjects, indicating that the flanker-induced changes were contingent upon the continued presentation of flankers.

## Introduction

Perceptual learning (PL) refers to a long-lasting improvement in one's perceptual abilities, which occurs with repeated exposure to the relevant stimuli during a period of training on a perceptual task [1]–[6]. Previously, we demonstrated that PL takes place in a contrast discrimination (CD) task in adult macaques [1], using a non-roving paradigm. Briefly, this involved a comparison of contrast levels between two consecutively presented stimuli per trial, where the contrast of the stimulus presented in the first interval (the ‘sample’) was fixed at 30% across trials, whereas the contrast of the stimulus presented in the second interval (the ‘test’) varied from trial to trial. We observed improvements in contrast discrimination performance over the course of training, indicating that it was possible- even in adult primates with well-developed visual perception- to hone their ability to make fine contrast discriminations. This corroborates reports from the human psychophysics literature, which show that healthy adult humans show similar improvements due to training in a CD task [2], [3], [7]–[10].

Additionally, human studies have explored PL under a variety of different task conditions, through 1) the implementation of a roving paradigm and/or 2) the addition of flanker stimuli. In a roving task (task manipulation #1 of this study), stimulus properties are allowed to vary unpredictably from trial to trial during *both* intervals, such that neither stimulus contrast is predictable between consecutive trials. Perceptual learning is possible under roving conditions, albeit to a limited extent [2], [3], and the pace of learning is influenced by the temporal structure of stimulus presentation [8], [9]. High levels of stimulus uncertainty make a task harder to learn, and therefore performance improvements are slower, diminished, or sometimes absent altogether [2], [3], [9], [11], [12]. It has been hypothesized that a roving paradigm impairs task performance through the continual disruption of memory traces [2], [9], thereby preventing observers from constructing and maintaining internal reference templates of stimulus contrasts.

Flanker stimuli (task manipulation #2) are often used to explore the role of context-dependent neural plasticity in perception and learning. A number of studies have argued that the addition of flanker stimuli changes the balance of excitation and inhibition in a local network and therefore allows for increased plasticity, which then yield improved perceptual learning in adults [3], [13]–[15] (but see Yu et al. [2]). A human psychophysics study by Adini et al. (2002) [13] examined the effects of flanker training on CD thresholds, and found that while training with flankerless stimuli produced no significant improvement, the presence of flanker stimuli yielded threshold reductions of ∼50%. Similarly, Tsodyks et al. (2004) [14] and Adini et al. (2004) [3] reported flanker-induced reductions in threshold on a CD task, as well as facilitations of task performance that varied depending on the length of flanker stimuli. To date, the effects of flankers have not been studied under a roving paradigm in macaque monkeys.

The aim of the current study was thus to expand our knowledge of the contrast discrimination capabilities of non-human primates, to better reflect the variety of tasks that have been carried out with human subjects. We investigated how the introduction of a roving task and the addition of flanker stimuli may affect perceptual learning of contrast discrimination. Given sufficient practice, would macaque subjects show improvements in CD? If so, would improvements occur across all sample contrast conditions, or for only a specific subset of conditions? If dramatic improvements proved possible, then we reasoned that an extension of the training period might yield similar benefits in human subjects (as with those observed by Parkosadze et al. [11] during a bisection task). On the other hand, if results were more uneven (mirroring those of Adini et al. [3] and Yu et al. [2]), then the lack of substantial improvement might be due to inherent challenges posed by the roving paradigm itself.

We found that partial improvements in CD occurred in the absence of flankers under roving stimulus conditions, when the sample contrast varied from trial to trial. The effects of adding flankers differed between subjects, with no long-lasting effects on performance after the removal of flankers, indicating that flanker-induced changes occurred only as long as flanker stimuli were present.

## Methods

All procedures were carried out in accordance with the European Communities Council Directive RL 2010/63/EC, the US National Institutes of Health Guidelines for the Care and Use of Animals for Experimental Procedures, and the UK Animals Scientific Procedures Act. The UK Home Office reviewed and approved this study (license PPL60/4037). Two male macaque monkeys (5–14 years of age; 10–13 kg) participated in these experiments. Subjects were housed in pairs or triplets in custom-built primate cages (Arrowmight), illuminated with natural and artificial light, and provided with environmental enrichment in the form of toys, nets, branches, and hidden treats. They were kept on a water restriction regime during weekdays, with free water access on weekends, and provided with a varied diet of fruit, nuts, and nutrient-enriched pellets, following the recommendations of the NC3Rs. Veterinary care and close monitoring by staff and technicians ensured prompt and effective interventions in the form of surgery, anaesthetics, antibiotics, and analgesics as needed, to maintain the health of the animals and minimise suffering. Both animals were sacrificed at the conclusion of the study with an overdose of pentobarbital, in compliance with the UK Home Office Codes of Practice.

### Stimuli

Stimulus presentation was controlled using CORTEX software (Laboratory of Neuropsychology, National Institute of Mental Health, http://dally.nimh.nih.gov/index.html) on a computer with an Intel Core i3-540 processor. Sinusoidal grating stimuli were displayed at a viewing distance of 0.54 m, on a 25″ Sony Trinitron CRT monitor with display dimensions of 40 cm (W) by 32 cm (H) and a resolution of 1280 by 1024 pixels, yielding a resolution of 31.5 pixels/degree of visual angle (dva). The monitor refresh rate was 85 Hz for monkey 1, and 75 Hz for monkey 2. The outputs of the red and green guns were combined using a Pelli-Zhang video attenuator [16], yielding a luminance resolution of 12 bits/pixel, allowing the presentation of contrasts that were well below contrast discrimination thresholds. A gamma correction was used to linearize the monitor output.

Unlike in the previous study by Chen et al. [1], the contrast of the sample stimulus was not fixed at 30%, but could take on one of three values (20, 30 or 40%) on a given trial. The test stimulus took on one of 12 possible contrasts, depending on the sample contrast (20% sample: [5, 10, 12, 15, 18, 22, 25, 28, 35, 45, 60, 90% test]; 30% sample: [5, 10, 15, 22, 25, 28, 32, 35, 38, 45, 60, 90% test]; 40% sample: [5, 10, 15, 25, 32, 35, 38, 42, 45, 50, 60, 90% test]), yielding 36 conditions in total.

Roving grating stimuli were positioned at parafoveal locations in the visual field, at the same lower hemifield location as that used in the non-roving task from the previous study, i.e. at an eccentricity of 4.6° (azimuth: −3.5°, elevation: −3°) and 1.5° (azimuth: −1.3°, elevation: −0.7°) for monkeys 1 and 2, respectively. Data were gathered in conjunction with the recording of neuronal data (not presented here), and the slight difference in stimulus location between the animals was due to a difference in the receptive field locations of the neurons that were sampled by the implanted electrodes. Gratings were vertically oriented; the SF was 4 cycles per degree (cpd) in both monkeys; and the diameter was 3 dva in monkey 1 and 0.75 dva in monkey 2. Apart from the contrast levels, all stimulus parameters were the same as those used previously during training on the non-roving task described in Chen et al. [1].

During the phase of training involving flanker stimuli, flanker gratings were displayed collinearly immediately above and below the central sample and test stimuli, forming a column of three gratings, positioned edge to edge. The flanker stimuli were identical to the sample and test stimuli in terms of size, SF and orientation. To optimise our chances of success under flanker conditions, we followed Adini et al.'s paradigm [3], using chains of flankers (rather than the elongated Gabors used by Yu et al. [2]) and kept the contrast of flankers constant at 30% throughout training, regardless of the sample contrast. However, we continued to vary the sample contrast from trial to trial (even though Adini et al. [3] reported better results for a blocked than for a ‘mixed by trial’ (‘MBT’) method), because we wanted to keep our paradigm as similar as possible to that used in the previous stage of roving training and ensure a smooth transition to the flanker task for our monkeys.

In addition, monkey 2 participated in a control task, in which the stimulus properties and locations were identical to those used with monkey 1 (4.6° eccentricity; 4 cpd; 3 dva diameter).

### Contrast discrimination task paradigm

**Figure 1:**
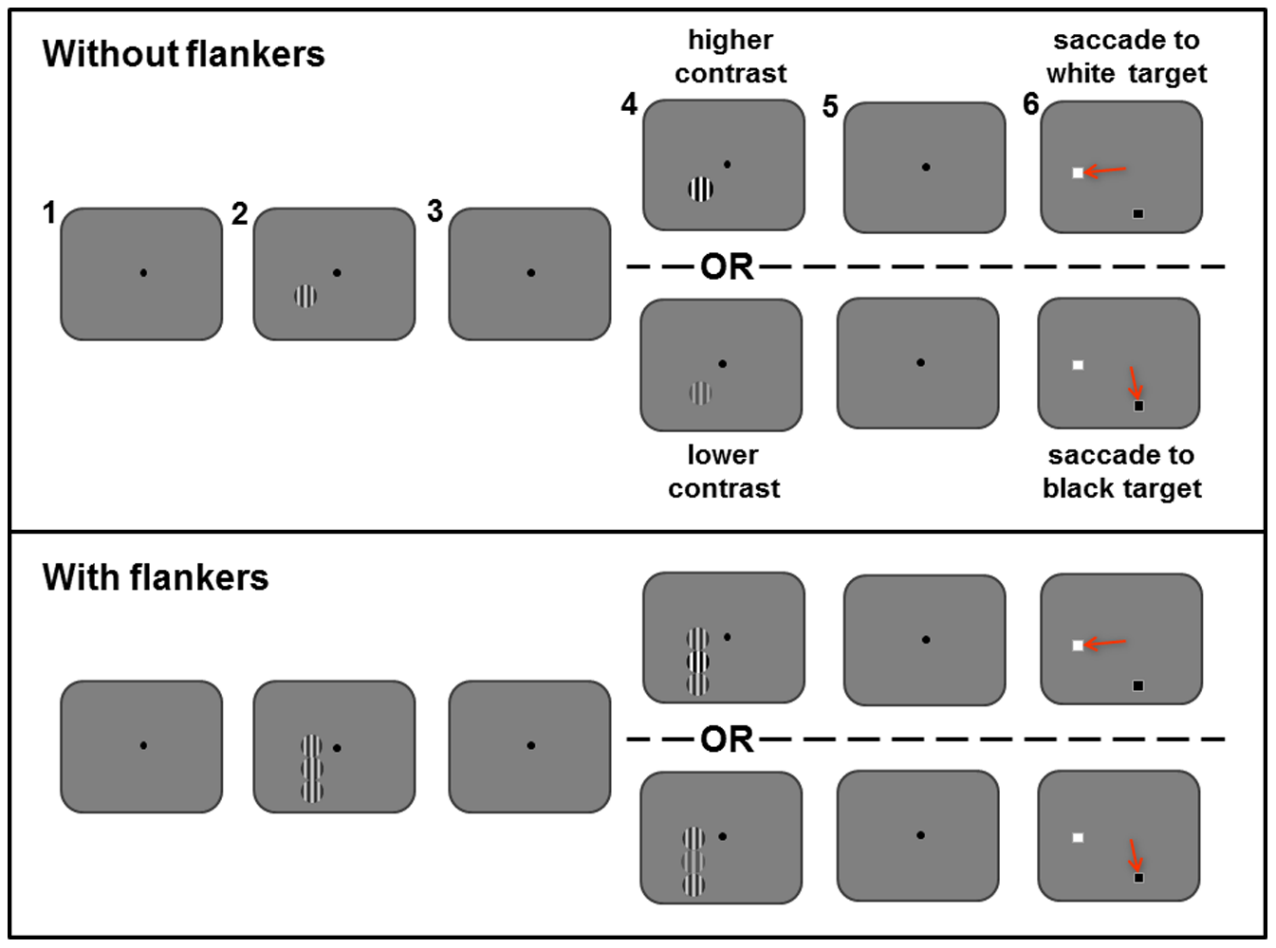
Illustration of the contrast discrimination task. Subjects performed the task in the absence (top) and then in the presence (bottom) of flanker stimuli. 1) The monkeys were required to fixate upon a central spot, to initiate the trial. 2) While maintaining fixation, a sample stimulus (in the form of a sinusoidal grating of 20%, 30% or 40% contrast) was presented for 512 ms. 3) Presentation of the sample was followed by an interval lasting 512 ms. 4) Next, the test stimulus (which could be of higher or lower contrast than the sample), was presented for 512 ms, 5) followed by a second interval of 400 ms. 6) Two target stimuli were presented to the left and right of the location at which the sample and test had previously appeared; the fixation spot changed colour from black to grey, signalling that the animals were allowed to make a saccade to their chosen target. If the test was of a higher contrast (e.g. 32%) than the sample (e.g. 30%), the monkeys had to saccade to the white target; otherwise, if the test stimulus was of a lower contrast (e.g. 28%), they had to saccade to the black target. The red arrows in the figure indicate the direction of saccadic motion for illustrative purposes only; they did not appear onscreen.

During training on the CD task, the presentation of a sample stimulus was followed by that of a test stimulus, and subjects had to decide whether the test stimulus was of higher or lower contrast than that of the sample (see Figure 1 for an illustration of the task). If the test stimulus was of lower contrast than the sample, the monkey had to saccade to a black target, otherwise it had to saccade to a white target. These basic requirements of the CD task were identical to those used previously during training on the non-roving task (described in Chen et al. [1]).

**Figure 2:**
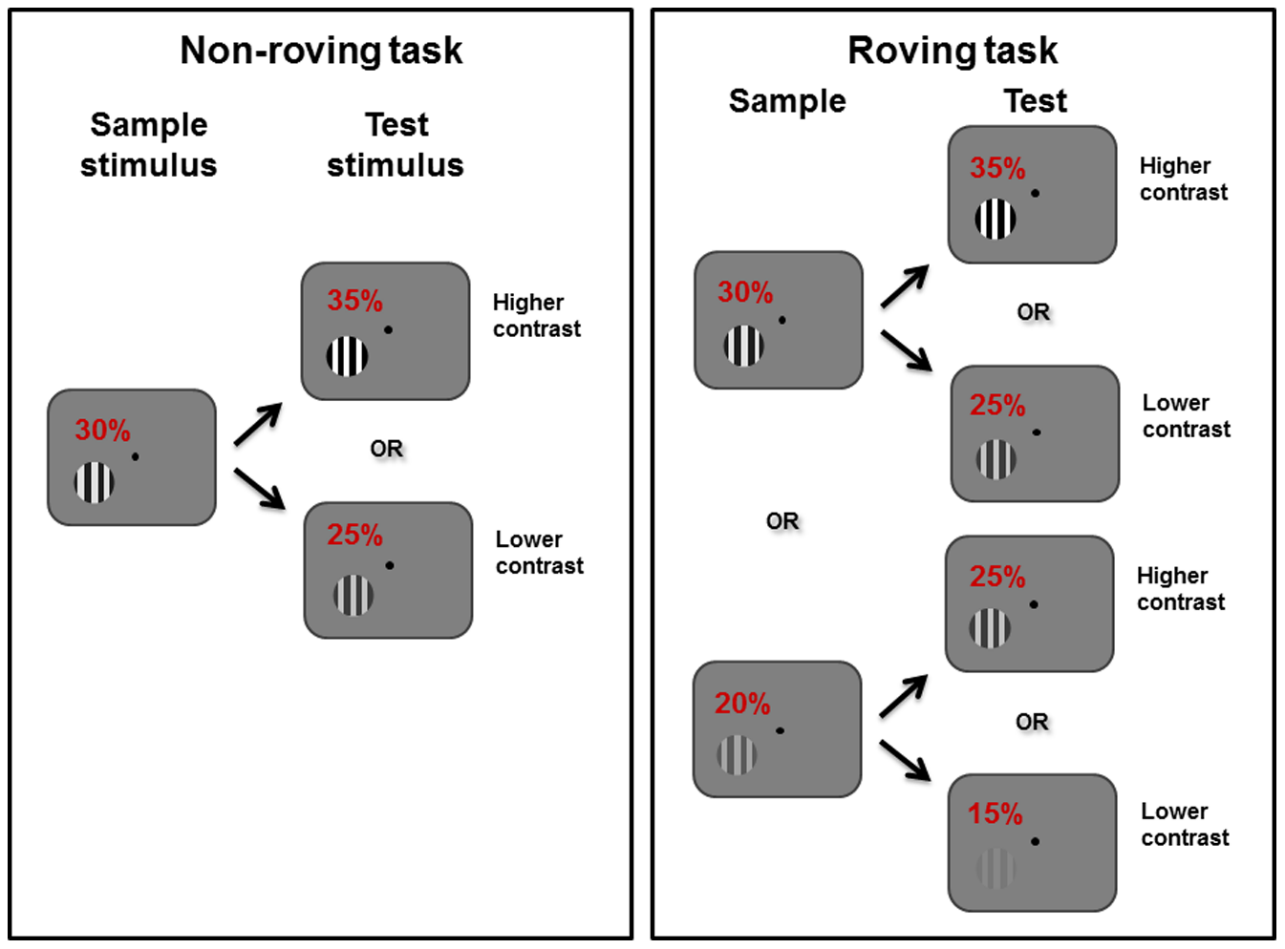
Characteristics of tasks involving non-roving and roving stimuli. Left panel: in tasks with non-roving stimuli, as in Chen et al. [1], the sample stimulus was always displayed at a contrast of 30%. Right panel: for the task in the current study, involving roving stimuli, the contrast of the sample stimulus varied randomly from trial to trial and took on a value of 20%, 30% or 40%. Unlike in the non-roving task, subjects had to take note of the contrast of the sample stimulus in order to perform the roving task correctly. For example, for a test stimulus of 25% contrast, they were required to make a saccade to the white target if it had been preceded by a sample of 20% contrast. On the other hand, they were required to make a saccade to the black target if the sample contrast had been 30% or 40%. Note that the contrasts of stimuli in the diagram are exaggerated for illustrative purposes.

For certain conditions, the identity of the correct target was the same regardless of the sample contrast (e.g. when the test contrast was 5%, the sample contrast was always higher, thus subjects always had to saccade to the black target). However, for other conditions (termed ‘response conflict conditions’), the identity of the correct target varied, depending on the sample contrast. For example, when the test contrast was 25%, if the sample contrast had been 30% or 40%, then the subjects had to saccade to the black target, whereas if the sample contrast had been 20%, the subjects had to saccade to the white target (refer to Figure 2 for an illustration of sample-dependent or sample-independent task requirements).

### Stages of training

Psychometric performances of the two subjects on the roving contrast discrimination task were monitored throughout the training process to allow a continuous assessment of behavioural improvement, across a total of 55 and 42 sessions for monkeys 1 and 2, respectively.

Training on the roving task was initially carried out in the absence of flankers (monkey 1: 33 sessions, spanning 8 weeks; monkey 2: 16 sessions, spanning 4 weeks). Unlike in previous human studies, we could not explicitly instruct our monkeys to base their decisions on comparisons between the sample and test stimuli, and disregard the rules learnt during non-roving training (i.e. the instruction to always make a comparison against a reference contrast of 30%). Thus, a fairly long training period was required, in which subjects obtained feedback via reward delivery, which shaped their understanding of the task requirements.

Once the subjects' performance had plateaued and it seemed unlikely that additional training would bring about further improvement, flanker stimuli were added, and training resumed in the presence of flankers (monkey 1: 15 sessions, spanning 6 weeks; monkey 2: 22 sessions, spanning 6 weeks). Finally, the flankers were removed and training continued in the absence of flanker stimuli (monkey 1: 7 sessions, spanning 1.5 weeks; monkey 2: 4 sessions, spanning 1 week).

### Measures of perceptual learning

To investigate the effects of perceptual learning on a stimulus roving task, several metrics of performance were used over the course of training: the proportion of correct responses made by the subjects (‘*P_correct_*’); the slope and the point of subjective equality (PSE) of the psychometric function; the psychometric threshold; the rate of learning for different contrasts; and the subjects' reaction times. For derivations of each of these measures, please refer to Chen et al. [1] for details.

### Calculation of Akaike's Information Criterion (AIC) values

During roving training under response conflict conditions, one would expect learning to be accompanied by a divergence in the monkeys' responses, depending on the sample contrast that was presented. Alternatively, if no learning occurred, then one would not expect sample-dependent differences in responses to emerge. A simple binomial test would be able to detect a difference in performance levels between sample contrast conditions (e.g. if it was conducted on the last third of training sessions); however, this might be the case even if little learning had occurred. In the event that our subjects' performance levels had already been high from the beginning of training on the roving task, then a binomial test would detect a difference between the roving conditions, but fail to indicate whether an improvement in performance had occurred over the course of training. Hence, we used a more complex approach which examined potential changes due to learning, in which we asked whether performance under roving conditions diverged with training (as would be expected if learning had occurred). We determined whether the data obtained under response conflict conditions were better described by a single (linear) model, or whether they were better described by separate linear models (and thus with two additional free parameters). To compare the two different models, an *AIC* value was calculated for each model, according to
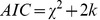
(Equation 1)where χ^2^ is the Chi-Square goodness of fit statistic (with an assumed variance of 1) and *k* is the number of free parameters in the model [17]. For the model involving two separate linear fits to the data (one fit to each half of the data, which were divided by sample contrast), *k* was equal to 4; for the model involving a single fit to the combined data, *k* = 2.

The *AIC* values were compared between the two models, in which a lower *AIC* corresponded to the model that provided a better description of the observed data. The Akaike model weight, *w_i_*, was calculated as a measure of the weight of evidence in favour of a particular model, as
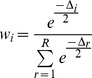
(Equation 2)where *i* is the model being evaluated; Δ*_i_* is the difference in *AIC* values between model *i* and the best model (i.e. the model with the lowest *AIC*); and Δ*_r_* is the difference in *AIC* values between model *r* and the best model, for the set of *R* models (in this case, *R* = 2). The larger the value of *w_i_*, the higher the relative likelihood of model *i*.

### Corrections for multiple comparisons

For all tests of significance that involved multiple comparisons, a False Discovery Rate (FDR) correction for α-levels was applied where appropriate, to reduce the likelihood of making either too many false positives or too many incorrect rejections [18]. This procedure yielded a ‘*q*-value,’ as an FDR analogue to the *p*-value.

## Results

### Learning the underlying requirements of the roving task

Previously, we reported changes in performance during perceptual learning of a CD task involving non-roving stimuli [1]. Following extensive training on the non-roving task, subjects were taught to perform the roving task, through reward feedback and conditioning. In order to assess how the roving paradigm initially affected our subjects' performance, parameters of learning were compared between non-roving and roving periods, thus part of the dataset from the earlier study is repeated here.

For certain conditions under the roving paradigm, the sample-target stimulus comparison varied depending on the sample contrast; these are termed ‘response conflict conditions.’ Namely, when the sample contrast was 20%, the conditions that induced a conflict (relative to the previously learned non-roving conditions where the sample was 30%) were those where the test contrast was lower than 30%, but higher than 20% (i.e. test contrasts of 22, 25 and 28%). When the sample contrast was 40%, the response conflict conditions were those where the test contrast was higher than 30%, but lower than 40% (i.e. test contrasts of 32, 35 and 38%).

**Figure 3:**
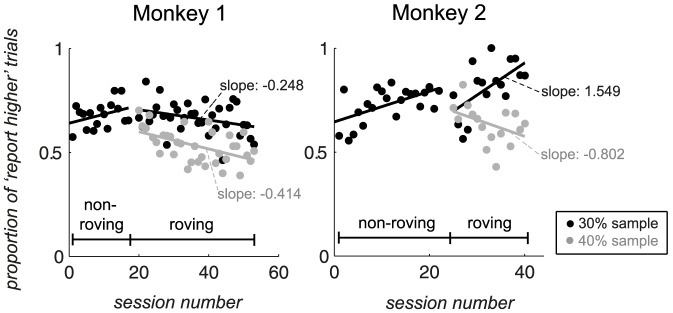
Proportion of ‘report higher’ trials (*P_reporthigher_*) against session number, for the condition where the test contrast was 35%. This condition demanded different responses, depending on the sample contrast (termed a ‘conflict condition’). Within each subplot, the leftmost data points indicate subjects' performance during the non-roving task, while those to the right indicate performance during the roving task. The sample contrast was 30% (black markers) or 40% (grey markers). A divergence in data points between response conflict conditions indicated that learning occurred under roving conditions.

In Figure 3, subjects' responses are plotted across roving and non-roving training periods, for an example response conflict condition (with a test contrast of 35%). The proportion of trials for which the subject reported the test contrast as being higher than the sample contrast is referred to as the proportion of ‘report higher’ trials (*P_reporthigher_*). *P_reporthigher_* for a sample contrast of 30% (black markers, Figure 3) is depicted alongside that when the sample contrast was 40% (grey markers). A visual comparison revealed that at the beginning of training on the roving task, monkey 1's responses to a given test contrast tended to be similar to that seen during the non-roving task, regardless of the actual contrast of the sample, i.e. he still based his judgment on the 30% reference that was used during the non-roving task. As training on the roving task continued, however, his responses diverged according to the contrast of the sample (indicated by a separation between black and grey markers over time), indicating that he learnt to carry out the roving task successfully. Similarly, in monkey 2, when roving stimuli were first introduced, the proportion of ‘report higher’ responses tended to overlap between the 30% and 40% sample conditions. However, responses gradually diverged over the course of roving training, showing that the monkey learnt to base his comparison on the sample contrast. Additionally, the divergence between data points was larger for monkey 2 than for monkey 1, indicating that learning was more pronounced in monkey 2 for this particular condition.

This divergence of responses, between competing sample contrast conditions, was seen for most of the six conflict conditions (Figure S1). To quantify how well subjects' responses could be separated based on the sample contrast, response conflict data were fitted with two separate linear models (as shown in Figure 3), as well as with a single linear model. To determine which model yielded a better description of the data, the *AIC* was calculated based on the joint value of the χ^2^ goodness of fit statistic from the two linear fits (yielding an *AIC* value for the ‘separate fittings’ model), and the *AIC* was calculated based on the χ^2^ statistic from the single-model linear fit (yielding an *AIC* value for the ‘single fitting’ model). Finally, the two *AIC* values were compared.

In all 12 cases, when separate fittings were carried out for the two halves of the data, the *AIC* value was smaller than that generated by a single fitting using the combined data, i.e. after additional free parameters had been accounted for, the separate fitting procedure yielded a better fit than the combined fitting procedure. This indicated that subjects' responses could be categorised into two distinct groups, according to the sample contrast. In each case, the value of *w_i_* was 1.00 for the model with separate fits (i.e. it was close to 0 for the model with a single fit), indicating that the relative likelihood of the ‘separate fittings’ model was consistently greater than that of the ‘single fitting’ model.

In addition, the slope of the best-fit line to the data was examined for each sample contrast condition, to provide a measure of the amount of change that occurred during training on the roving task (Table 1). One would expect that if the subjects failed to heed the sample contrast, then the slopes would be similar across sample contrasts. On the other hand, if they modified their behaviour over the course of training and learnt to heed the sample contrast, then the proportion of trials in which they reported a higher test contrast would change and ultimately differ, and this would be reflected as a difference in the slopes of the best-fit lines between sample contrast conditions.

**Table 1:** Slopes of the best-fit line to the roving data, shown in Figure 9 and Figure S1, for each response conflict condition.

Test contrast (%)		22	25	28	32	35	38
Slope	Sample contrast (%)						
		Monkey 1					
	20	0.08	−0.25	−0.204	-	-	-
	30	0.0148	−0.234	−0.436	−0.179	−0.248	0.00881
	40	-	-	-	−0.521	−0.414	−0.398
		Monkey 2					
	20	0.914	1.83	1.359	-	-	-
	30	0.85	0.761	0.415	0.328	1.549	−0.266
	40	-	-	-	0.0333	−0.802	−0.393

In the majority of cases, responses diverged with training on the roving task, according to the contrast of the sample.

In 11/12 cases, responses diverged between the two sample conditions, over the course of roving training. This indicated that subjects learnt to adjust their behaviour as required. For the one case in which no divergence occurred (monkey 1, test contrast of 25%), the slopes of the best-fit lines for the two sets of data (corresponding to sample contrasts of 20% or 30%) were similar (0.250 and 0.234, respectively) and the intercepts of the best-fit lines were relatively far apart in value (45.9 and 33.8, respectively), indicating that for this test contrast, the subject's performance was already high at the onset of roving training.

In summary, these results indicate that subjects learnt to heed the sample during training under roving conditions. Note that this portion of the analysis was not intended as a demonstration of perceptual learning of contrast discrimination per se, but rather, as evidence that the macaques were able to adjust their previous conception of the CD task (from a non-roving paradigm to a roving one), and that they learnt to carry out their comparisons between stimuli correctly during the roving task.

### Perceptual learning averaged across the hardest test contrast conditions

Since subjects had already undergone extensive training during the non-roving task, we hypothesised that learning would be most apparent for the response conflict conditions, whereas it would have already have reached asymptotic levels for the easy conditions. To obtain an overview of the degree of improvement attained for the most difficult test contrasts, *P_correct_* was calculated based on subjects' performance during conflict conditions only.

**Figure 4:**
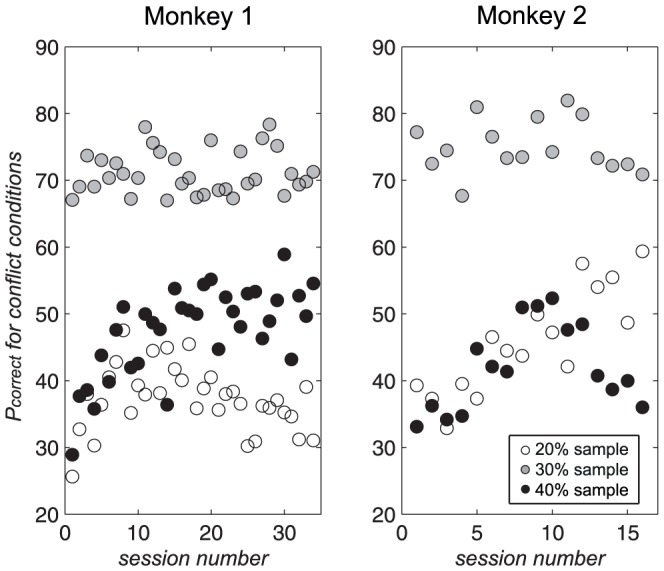
*P_correct_* (calculated solely based on response conflict conditions), as a function of session number.

To obtain *P_correct_*, the mean proportion of correct trials was taken across all the conflict conditions for each day, for each sample contrast condition. *P_correct_* for the response conflict conditions was then plotted as a function of session number (Figure 4), for the pre-flanker training period. To assess whether performance improved with training, a Mann-Whitney U test was carried out between the first and second half of sessions for each sample contrast, with an FDR correction for multiple comparisons. Improvements were seen for both subjects, for the 40% sample contrast in monkey 1, indicated by the negative values of the *Z* statistic (20%: *U*(32) = 360, *Z* = 2.135, *p* = .0327; 30%: *U*(32) = 303, *Z* = 0.172, *p* = .863; 40%: *U*(32) = 208, *Z* = −3.065, *p* = .00217, α = .05/3*2 = .0333), and the 20% sample contrast in monkey 2 (20%: *U*(14) = 39, *Z* = −2.993, *p* = .00276; 30%: *U*(14) = 68, *Z* = 0.0, *p* = 1.0; 40%: *U*(14) = 55, *Z* = −1.313, *p* = .189, α = .05/3 = .0167). (Note that a significant worsening was also observed for monkey 1 for the 20% sample, but this could be explained by the upside-down ‘U’ shape in the subject's performance for this sample contrast.)

### Perceptual learning averaged across all test contrast conditions

**Figure 5:**
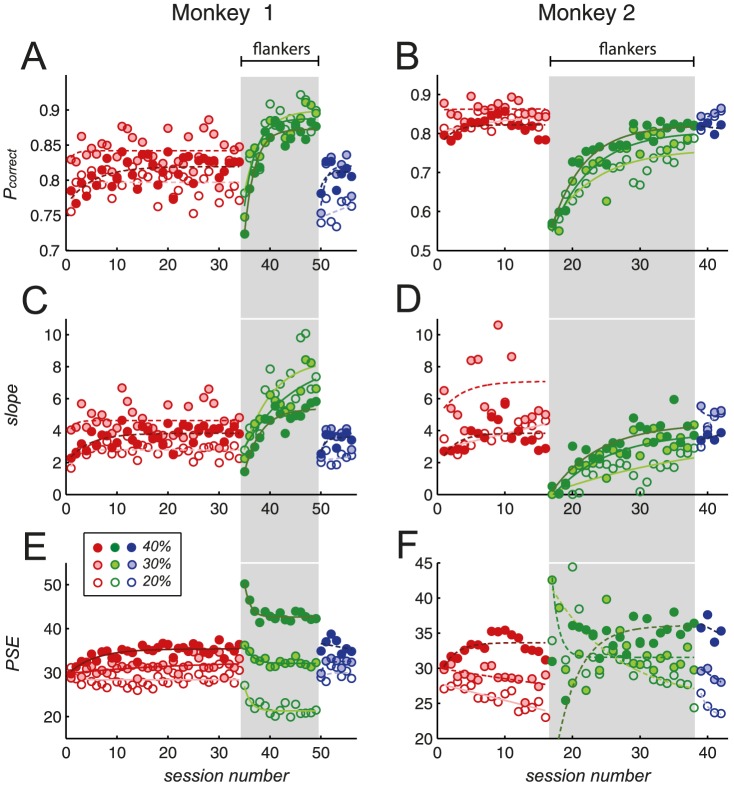
Measures of performance of the two subjects during the roving task. Left column: monkey 1; right column: monkey 2. A & B: *P_correct_*; C & D: slope of the psychometric function; E & F: PSE of the psychometric function. Red markers: pre-flanker training; green markers (grey background): flanker training; blue markers: post-flanker training. Unfilled markers: 20% sample contrast conditions; medium-coloured filled markers: 30%; dark-coloured filled markers: 40%.

Next, rates of learning were examined across all 12 test contrast conditions for each sample contrast, using three measures of performance for each session: 1) the mean proportion of correct responses, 2) the slope, and 3) the PSE of the psychometric curve. These are depicted by the red markers in Figure 5 (green and blue markers will be referred to in later sections).

To identify learning-induced changes, task performance was compared between the first and last 30% of sessions for each sample contrast, using a Mann-Whitney *U* test. Improvements were indicated by increases in the proportion of correct responses; increases in slope; and/or shifts in the PSE towards the sample contrast. For monkey 1, when the sample stimulus had a contrast of 40%, performance improved significantly across all three measures (Table 2, Mann-Whitney *U* test). For monkey 2, when the sample stimulus had a contrast of 20%, significant improvements were seen in the proportion of correct responses, the slope, and the PSE. In summary, during the roving task, improvements occurred for a subset of sample contrasts, but not for others, and results varied, depending on the subject.

**Table 2:** Comparisons of performance levels between early and late sessions during training with roving stimuli, using Mann-Whitney *U* test.

Monkey	1					2				
	Pre-flankers									
	*df* = (1,18)					*df* = (1,6)				
Statistic	μ_early_ (σ^2^_early_)	μ_late_ (σ^2^_late_)	*U*	*Z*	*q*	μ_early_ (σ^2^_early_)	μ_late_ (σ^2^_late_)	*U*	*Z*	*q*
	20%									
*P_correct_*	79.4 (3.5)	79.2 (5.0)	114	0.643	.521	81 (0.4)	83.8 (3.1)	10	−2.165	.0304*
Slope	2.7 (0.2)	2.7 (0.2)	110	0.340	.734	3.0 (0.2)	4.2 (0.1)	10	−2.165	.0304*
PSE	28.3 (2.1)	29.6 (4.9)	83	−1.625	.104	27.4 (0.2)	24.4 (0.4)	26	2.165	.0304*
*RT_correct_*	149.3 (125.5)	112.6 (53.1)	155	3.742	<.001*	165.9 (10.3)	162.5 (18.0)	24	1.588	.112
*RT_error_*	154.7 (94.1)	127.4 (128.6)	150	3.364	<.001*	169.5 (16.1)	169.8 (14.0)	20	0.433	.665
	30%									
*P_correct_*	83.8 (2.1)	84.4 (8.8)	98	−0.491	.623	85.1 (4.1)	85.7 (3.4)	18	0	1
Slope	4.5 (0.3)	4.7 (1.4)	104	−0.038	.970	5.2 (1.1)	7.2 (21.2)	20	0.433	.665
PSE	30.4 (1.2)	31.9 (3.3)	78	−2.003	.0452	29.6 (0.6)	27.3 (2.5)	25	1.876	.0606
*RT_correct_*	149.8 (114.7)	111.5 (40.3)	155	3.742	<.001*	165 (7.0)	162.7 (23.9)	23	1.299	.194
*RT_error_*	152.9 (98.1)	126.2 (96.7)	153	3.591	<.001*	171.1 (57.2)	166.3 (10.7)	23	1.299	.194
	40%									
*P_correct_*	79.6 (3.4)	82.1 (1.6)	71	−2.532	.0113*	79.6 (2.1)	80.8 (2.9)	17	−0.144	.885
Slope	3.1 (0.3)	3.9 (0.2)	64	−3.062	.00220*	2.8 (0.0)	3.4 (0.2)	12	−1.588	.112
PSE	32.9 (2.5)	35.3 (1.5)	64	−3.062	.00220*	31.5 (0.8)	33.0 (0.8)	15	−0.722	.470
*RT_correct_*	148.8 (123.8)	113.2 (52.3)	155	3.742	<.001*	165.4 (42.4)	163.2 (33.0)	21	0.722	.470
*RT_error_*	149.5 (123.3)	111.2 (180.4)	154	3.666	<.001*	168.2 (6.3)	160.8 (30.9)	26	2.165	.0304

	Flankers									
	*df* = (1,6)					*df* = (1,10)				
Statistic	μ_early_ (σ^2^_early_)	μ_late_ (σ^2^_late_)	*U*	*Z*	*q*	μ_early_ (σ^2^_early_)	μ_late_ (σ^2^_late_)	*U*	*Z*	*q*
	20%									
*P_correct_*	81.3 (4.8)	90.1 (5.5)	10	−2.165	.0304*	62.2 (17.5)	75.8 (6.5)	21	−2.802	.00507*
Slope	3.7 (0.4)	8.5 (3.0)	10	−2.165	.0304*	0.4 (0.3)	2.2 (0.3)	21	−2.802	.00507*
PSE	23.9 (5.0)	20.9 (0.1)	26	2.165	.0304*	38.3 (77.8)	27.5 (2.5)	57	2.802	.00507*
*RT_correct_*	128.3 (203.0)	126.2 (454.5)	19	0.144	.885	157.4 (6.9)	161.1 (20.6)	31	−1.201	.230
*RT_error_*	133.3 (357.7)	132.6 (228.0)	19	0.144	.885	162.5 (12.9)	170.2 (13.4)	22	−2.642	.00824
	30%									
*P_correct_*	81.6 (21.1)	90.0 (1.0)	10	−2.165	.0304*	63.8 (59.5)	79.2 (6.1)	21	−2.802	.00507*
Slope	3.8 (1.2)	7.4 (1.1)	10	−2.165	.0304*	1.0 (0.7)	3.4 (0.3)	21	−2.802	.00507*
PSE	34.1 (2.7)	31.8 (0.2)	26	2.165	.0304*	32.9 (39.5)	31.1 (1.4)	39	0.000	1
*RT_correct_*	126.8 (198.7)	119.8 (384.9)	20	0.433	.665	157.1 (6.2)	162.5 (11.3)	25	−2.162	.0306
*RT_error_*	133.2 (191.2)	132.6 (451)	18	0.000	1	158.4 (8.6)	162.3 (10.9)	28	−1.681	.0927
	40%									
*P_correct_*	78.5 (18.6)	87.2 (1.1)	10	−2.165	.0304*	66.3 (65.1)	82.1 (0.4)	21	−2.802	.00507*
Slope	2.6 (0.8)	5.5 (0.2)	10	−2.165	.0304*	1.5 (1.6)	4.2 (0.9)	21	−2.802	.00507*
PSE	45.5 (14.0)	42.7 (0.6)	22	1.010	.312	25.4 (342.0)	35.9 (1.3)	30	−1.361	.173
*RT_correct_*	125.5 (262.7)	123.3 (430.5)	19	0.144	.885	161.5 (1.9)	163.4 (16.1)	35	−0.560	.575
*RT_error_*	132.3 (214.8)	129 (387.8)	19	0.144	.885	154.5 (26.5)	157.3 (35.3)	34	−0.721	.471

Results are presented separately for pre-flanker training (left half of the table) and for flanker training (right half). An FDR correction for α-levels was performed for multiple comparisons due to the measures of performance under assessment (pre-flanker training, monkey 1: 20%: α = .05×1/3 = .0167; 30%: α = .05×1/3 = .0167; 40%: α = .05×3/3 = .05; pre-flanker training, monkey 2: 20%: α = .05×3/3 = .05; 30%: α = .05×1/3 = .0167; 40%: α = .05×1/3 = .0167; flanker training, monkey 1: 20%: α = .05×3/3 = .05; 30%: α = .05×3/3 = .05; 40%: α = .05×2/3 = .0333; flanker training, monkey 2: 20%: α = .05×3/3 = .05; 30%: α = .05×2/3 = .0333; 40%: α = .05×2/3 = .0333). For changes in reaction time, an FDR correction was carried out to take the two measures of RT, *RT_correct_* and *RT_error_*, into account (pre-flanker training, monkey 1: 20%, 30% & 40%: α = .05×2/2 = .05; pre-flanker training, monkey 2: 20%%, 30% & 40%: α = .05×1/2 = .025; flanker training, monkey 1: 20%%, 30% & 40%: α = .05×1/2 = .025; flanker training, monkey 2: 20%%, 30% & 40%: α = .05×1/2 = .025). Significant changes are indicated by an asterisk.

**Figure 6:**
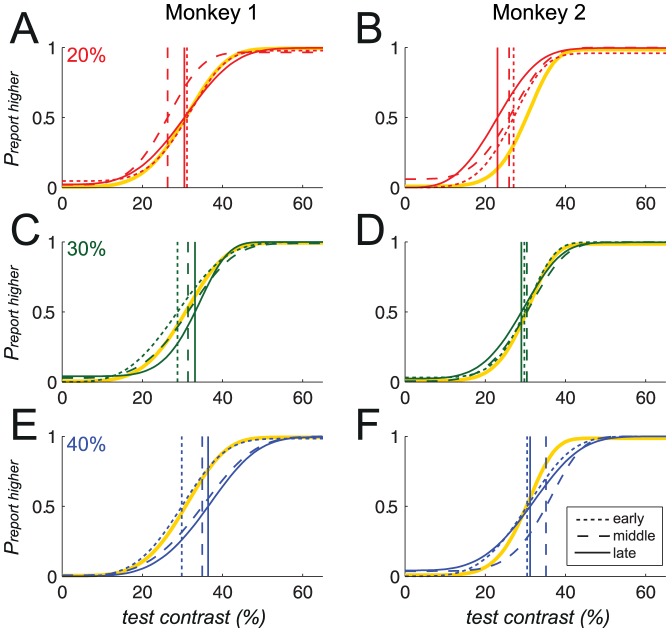
Illustration of changes in the psychometric function over the course of training. Plots show the psychometric functions obtained from three sessions of roving training, namely from the first (dotted line), middle (dashed line), and last sessions (solid line), as well as during the final session of non-roving training (yellow line). *P_reporthigher_* was plotted against the test contrast, and data were fitted using a Weibull function. Lines represent the fitted curve. Left column: monkey 1; right column: monkey 2. A & B: 20% sample (red); C & D: 30% sample (green); E & F: 40% sample (blue). Improvement in the psychometric function is represented by a shift in the PSE (vertical line) towards the respective sample contrasts; such a shift is clearly visible for the 40% sample condition in monkey 1, and the 20% sample condition in monkey 2.

This is additionally demonstrated in Figure 6, which plots the psychometric functions obtained during example sessions, at the start (dotted line), middle (dashed line), and end (solid line) of roving training. The psychometric function obtained during the last non-roving session (yellow line) is also plotted on each of the graphs, for comparison. Improvements in the psychometric function are represented as shifts in the PSE (vertical lines) towards the respective sample contrasts, and are clearly seen for the 40% sample condition in monkey 1, and the 20% sample condition in monkey 2.

### Relative changes in performance based on sample contrast

**Figure 7:**
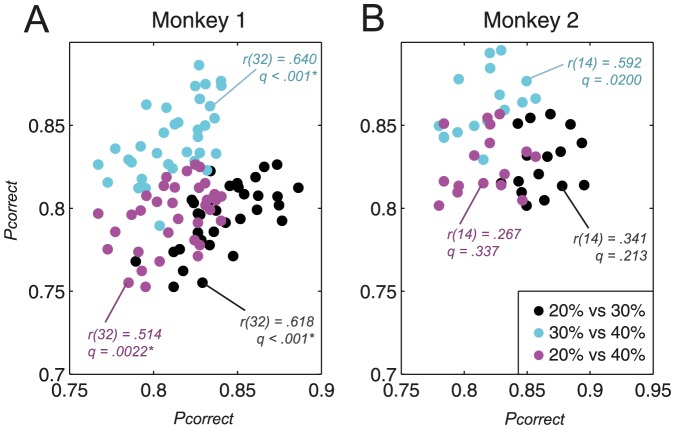
*P_correct_* for each pairwise comparison between sample contrasts. A: monkey 1; B: monkey 2. 20% versus 30%: black; 30% versus 40%: cyan; 20% versus 40%: magenta.

In theory, these sample-specific improvements in performance could simply have originated from a shift in the decision criterion chosen by the subjects, causing subjects to favour one response over another. Such a bias would then translate into an apparent ‘improvement’ for a particular sample contrast, but be accompanied by poorer performance for a different sample contrast. If so, then performance levels would be negatively correlated between pairs of sample contrasts. In order to remove the potentially confounding effect of task learning, a partial correlation was performed, in which we controlled for session number (Figure 7).

Contrary to the above prediction, the proportions of correct trials were significantly *positively* correlated (Spearman's partial rank-order correlation) for each of the three comparisons made in monkey 1; correlations were also positive (though not significant) in monkey 2 (FDR correction for multiple comparisons, α = .05/6×3 = .025). Thus, improvements for selected sample contrasts did not occur at the expense of performance on other sample contrasts, indicating that CD learning was genuinely responsible for the selective enhancements in performance, rather than a mere shift in criterion levels.

### Addition of flankers

Subjects practised a roving contrast discrimination task with flanker stimuli for several weeks, until their performance reached a plateau. As with the flankerless paradigm in the previous section, learning rates were monitored across all 12 test contrast conditions for each sample contrast, using three measures of performance for each session (green markers, Figure 5). Task performance was compared between the first and last 30% of flanker sessions, for each sample contrast, using a Mann-Whitney *U* test.

In both animals, from the beginning to the end of flanker training, the proportion of correct trials and the slope increased significantly across all three sample contrasts conditions. Furthermore, in monkey 1, the PSE shifted significantly towards the sample contrast, for sample contrasts of 20% and 30% (refer to Table 2), while in monkey 2, a shift of the PSE occurred towards the value of 20%, for the 20% sample contrast condition. Thus, during roving training in the presence of flanker stimuli, improvements occurred for both subjects, across all three sample contrast conditions. During the previous period of roving training, on the other hand- conducted in the absence of flankers- improvements had only occurred for a limited subset of sample contrast conditions.

### Comparison of performance before and after addition of flankers

An important question was whether the improvements seen within the flanker training period resulted in performance levels that surpassed those seen prior to the addition of flankers. A comparison of performance levels between pre-flanker and flanker training revealed that indeed, for monkey 1, the gains made during flanker training boosted his performance beyond that attained in the absence of flankers (left column, green versus red markers, Figure 5). Values of *P_correct_* and the slope were significantly higher at the end of flanker training, than at the end of pre-flanker training, for all three sample contrast conditions (monkey 1, 20% sample: *P_correct_*, *U*(12) = 50, *Z* = 2.758, *q* = .00582, slope, *U*(12) = 50, *Z* = 2.758, *q* = .00582; 30% sample: *P_correct_*, *U*(12) = 50, *Z* = 2.758, *q* = .00582, slope, *U*(12) = 49, *Z* = 2.616, *q* = .00889; 40% sample: *P_correct_*, *U*(12) = 50, *Z* = 2.758, *q* = .00582, slope, *U*(12) = 50, *Z* = 2.758, *q* = .00582, Mann-Whitney *U* test). Improvements in the PSE also occurred for sample contrasts of 20% and 40% (monkey 1, 20% sample: PSE, *U*(12) = 10, *Z* = −2.758, *q* = .00582; 30% sample: PSE, *U*(12) = 32, *Z* = 0.212, *q* = .832; 40% sample: PSE *U*(12) = 10, *Z* = 2.758, *q* = .00582, Mann-Whitney *U* test, FDR correction for α-levels, 20%: α = .05×3/3 = .05; 30%: α = .05×2/3 = .0333; 40%: α = .05×3/3 = .05).

The pattern observed in monkey 2's performance was markedly different (right column, Figure 5), as the introduction of flankers triggered a substantial drop in performance. As reported above, this monkey's performance improved during the flanker training period itself; however, for the 20% and 30% sample contrast conditions, these improvements were not sufficient to overcome the initial drop seen upon the addition of flankers. The *P_correct_* and slope showed significant improvement for the 40% sample, but were significantly worse at the end of flanker training than at the end of pre-flanker training for the 20% and 30% samples (monkey 2, 20% sample: *P_correct_*, *U*(8) = 34, *Z* = 2.452, *q* = .0142, slope, *U*(8) = 34, *Z* = 2.452, *q* = .0142, PSE, *U*(8) = 12, *Z* = −2.025, *q* = .0428; 30% sample: *P_correct_*, *U*(8) = 34, *Z* = 2.452, *q* = .0142, slope, *U*(8) = 34, *Z* = 2.452, *q* = .0142, PSE, *U*(8) = 10, *Z* = −2.452, *q* = .0142; 40% sample: *P_correct_*, *U*(8) = 12, *Z* = −2.025, *q* = .0428, slope, *U*(8) = 12, *Z* = −2.025, *q* = .0428, PSE, *U*(8) = 10, *Z* = −2.452, *q* = .0142, FDR correction for α-levels, 20%: α = .05×3/3 = .05; 30%: α = .05×3/3 = .05; 40%: α = .05×3/3 = .05). The PSE lay closer to the value of 40% for the 40% condition, but further away from the value of 30% for the 30% condition. Hence, for two of the three sample contrast conditions, this subject failed to improve beyond the peak levels that had been reached prior to flanker training.

### Removal of flanker stimuli

Finally, flankers were removed and subjects performed the roving task using isolated sample and test gratings, as was done before the introduction of flankers. This was to determine whether the flanker-induced changes would persist under flankerless conditions.

A visual inspection of subjects' performance upon the removal of flankers revealed that performance returned to pre-flanker levels (blue markers, Figure 5). We anticipated that the subjects' performance during the first few sessions following flanker removal might be relatively poor as they adjusted to the previous, flankerless version of the task. Thus, our analysis focused on data that were obtained from the last session of the second period of flankerless training (i.e. after flanker removal).

For the most part, subjects' performance during this session (*X*_a_) lay within the ranges of values seen during the late phase of the initial flankerless stage (Table 3, spanning nine sessions for monkey 1 and four sessions for monkey 2). For monkey 1, the proportion of correct responses, the slope, the PSE, *RT_correct_* and *RT_error_* lay within the ranges attained during the late phase of pre-flanker training for the 20% sample, while they were either within the ranges or slightly worse, for the 30% and 40% sample. For monkey 2, although *RT_correct_* and *RT_error_* were worse during the last flankerless session, values of the slope fell within previous ranges for the 30% and 40% samples, while for the 20% sample, the proportion of correct responses, the slope, and the PSE were slightly better than before.

**Table 3:** Comparison of subjects' performance in the absence of flankers, during post-flanker sessions, and during the end of pre-flanker sessions.

	Monkey 1		Monkey 2	
	Late pre-flanker sessions, range *X*_min_ – *X*_max_	Last post-flanker session, *X*_a_	Late pre-flanker sessions, range *X*_min_ – *X*_max_	Last post-flanker session, *X*_a_
	20% sample			
*P_correct_* (%)	75.2–82.5	76.3	81.6–85.4	85.7
Slope	2.0–3.1	2.4	3.7–4.5	5.1
PSE	27.7–34.8	28.7	23.8–25.3	23.5
*RT_correct_*	100.1–124.3	119.4	158.4–166.2	170
*RT_error_*	110.6–148.5	136.9	166.5–174.7	179.1
	30% sample			
*P_correct_* (%)	78.9–88.6	77.7	84.3–88.4	86.4
Slope	2.9–6.6	2.8	4.7–14.1	5.3
PSE	28.7–34.7	32.4	25.1–28.5	28
*RT_correct_*	103.0–118.9	120	157.3–167.4	170.2
*RT_error_*	113.5–143.7	131.6	163.1–170.8	175.4
	40% sample			
*P_correct_* (%)	79.5–83.3	80.5	78.2–82.1	82.2
Slope	3.2–4.3	3.4	2.8–4.0	3.9
PSE	33.1–37.4	34.8	32.4–34.3	35.3
*RT_correct_*	102.6–121.9	123.4	156.4–169.2	172.1
*RT_error_*	91.7–136.9	115.3	154.6–166.4	169.7

*X*_min_ – *X*_max_: Ranges of performance seen during late pre-flanker sessions, which took place before flankers were introduced. *X*_a_: Performance recorded during the last session of post-flanker training.

Thus, the monkeys' ability to discriminate contrast levels was largely similar when performance was compared before and after flanker training, indicating that any changes in performance that accompanied the addition of flankers were temporary and depended on the presence of flankers.

### Perceptual learning for individual test contrast conditions

**Figure 8:**
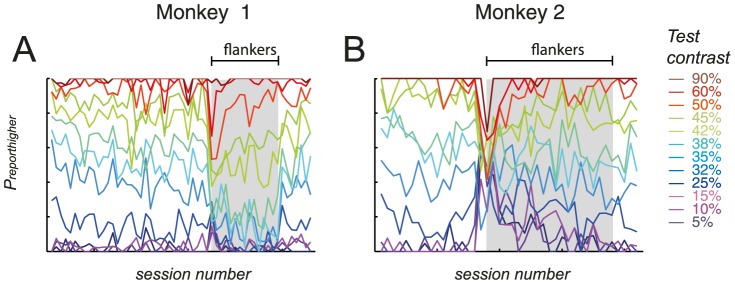
Proportion of ‘report higher’ trials (*P_reporthigher_*) against session number, for a 40%-contrast sample. Test contrast conditions are coded by colour. Training was initially carried out without flankers, then a period of flanker training commenced (grey background), followed by several sessions without flankers. A: monkey 1; B: monkey 2.

To examine how learning rates differed between test contrast conditions, performance was plotted separately for each of the test contrasts (Figure 8 and Figure S2).

The greater the difference between sample and test contrasts, the better the subjects' performance, and the faster an asymptotic level of performance was reached.

### Psychometric thresholds

To calculate psychometric thresholds, conditions were separated into two ‘test contrast categories,’ where the test contrast was (a) higher or (b) lower than the sample contrast (termed groups C_H_ and C_L_, respectively). For each group, performance levels were plotted against the absolute difference between the sample and test contrasts, and a Weibull curve was fitted to the data, yielding two thresholds, *T_L_* and *T_H_*, for conditions where the contrast of the test stimulus was lower and those where it was higher, respectively (refer to Chen et al. [1] for details). A Spearman's rank correlation analysis was carried out between threshold and session number, to test for changes in the threshold over time. During the pre-flanker training period, significant decreases in upper and lower threshold values were observed in monkey 1 for the 40% sample contrast and in lower thresholds in monkey 2 for the 20% and 30% sample contrasts (Table 4). These changes matched the selective improvements seen in subjects' performance and in the slope and PSE of their psychometric functions with training, i.e. predominantly for the 40% and 20% sample contrasts in monkeys 1 and 2 respectively.

**Table 4:** Changes in psychometric thresholds during the roving task, during the pre-flanker period as well as during the flanker period.

Statistic	*df*	*r*	*q*	*df*	*r*	*q*
	Monkey 1			Monkey 2		
	Pre-flankers					
	20%					
C_L_	32	−.107	.545	14	.765	<.001*
C_H_	32	.371	.0315	14	−.541	.0327
	30%					
C_L_	32	−.303	.0814	14	.359	<.001*
C_H_	32	.246	.160	14	−.406	.0327
	40%					
C_L_	32	−.429	.0120*	14	−.018	.952
C_H_	32	.428	.0115*	14	.356	.176

	Flankers					
	20%					
C_L_	13	−.304	.271	20	−.408	.0591
C_H_	13	−.682	.00653*	20	−.673	<.001*
	30%					
C_L_	13	−.609	.0159*	20	−.672	<.001*
C_H_	13	−.764	.00139*	20	−.810	<.001*
	40%					
C_L_	13	−.529	.0454	20	−.889	<.001*
C_H_	13	−.836	<.001*	20	−.692	<.001*

* *q*<α.

FDR correction for multiple comparisons, pre-flankers: α = .05/12×4 = .0167; flankers: α = .05/12×9 = .0375.

During flanker training, significant decreases occurred for all upper threshold values, as well as for the majority of lower thresholds (Table 4). These widespread improvements occurred across all three sample contrasts and thus matched those observed in the other parameters of performance. (Note that the addition of flankers induced changes in performance that occurred in opposite directions between the two monkeys, hence this involved a comparison of early versus late sessions of the flanker training, rather than a comparison of pre-flanker versus flanker sessions.)

### Reaction times

For each session, mean RTs were calculated separately for correct and incorrect trials, across all 12 test contrast conditions for each sample contrast. We investigated whether the mean RT changed over the course of training, within each epoch. In monkey 1, during pre-flanker training, RTs decreased significantly with training across all sample contrast conditions, for correct as well as for incorrect trials, while no improvement was observed during the period of training with flankers (Table 2). In monkey 2, no significant reduction in RT occurred during the pre-flanker stage, but RTs decreased for error trials when the sample contrast was 20%, during training with flankers (Table 2).

### Control task with matching locations between the subjects

During training on the roving task, reported thus far, the RF locations of the stimuli differed slightly between the two subjects (4.6° eccentricity in monkey 1 and 1.5° eccentricity in monkey 2). The modulatory effects of attention are eccentricity-dependent; for example, they are found to differ between parafoveal and peripheral visual field locations [19]. This thus raised the question of whether the divergent patterns of performance between the subjects, which occurred upon the introduction of flankers, could have resulted from a difference in stimulus eccentricity. To explore this possibility, an additional period of training was carried out, in which monkey 2 was presented with stimuli that were located at the same coordinates as those used for monkey 1, i.e. at 4.6° of eccentricity. His behavioural performance was monitored over a total of 60 sessions (23 pre-flanker sessions; 22 flanker sessions; and 5 post-flanker sessions).

**Figure 9:**
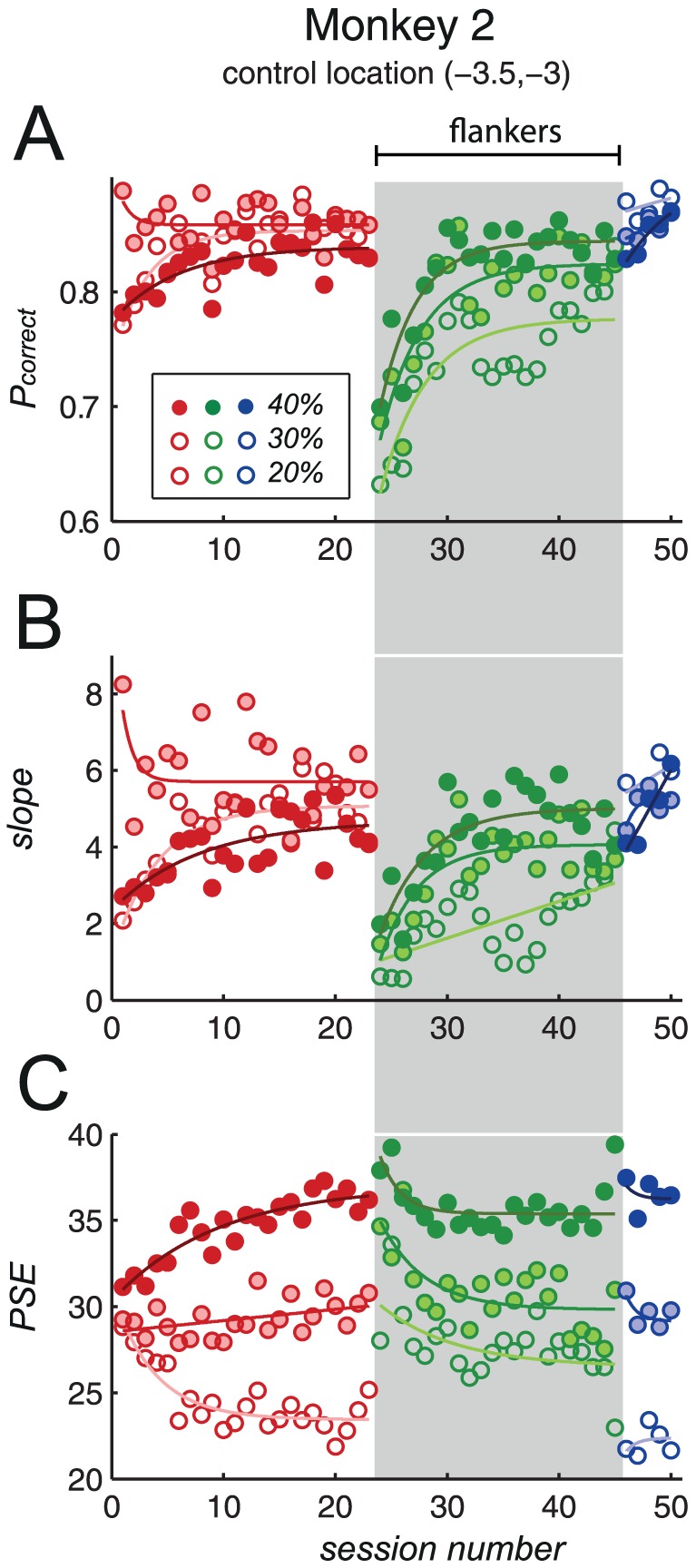
Monkey 2's performance, when stimulus eccentricity was identical to that used for monkey 1 (4.6°). The drop in performance upon addition of flankers, the gradual improvement during flanker training, and the subsequent return to pre-flanker levels, was similar to that previously seen with a stimulus eccentricity of 1.5°. A: *P_correct_*; B: slope of the psychometric function; C: PSE. Red data points: pre-flankers; green data points (grey background): flankers; blue data points: post-flankers. Unfilled markers: 20% sample contrast conditions; medium-coloured filled markers: 30%; dark-coloured filled markers: 40%.

The proportion of correct trials, the PSE and the slope of the psychometric function were plotted against session number (Figure 9). Results were similar to those seen previously in this monkey, when stimuli were presented at 1.5° eccentricity- uneven gains in performance during pre-flanker training were followed by a steep initial drop in performance when flankers were introduced; furthermore, despite marked improvement, performance levels during flanker training did not improve beyond those seen during pre-flanker training, and returned to pre-flanker levels upon the removal of flanker stimuli. We thus concluded that the differences in performance seen between the two subjects during pre-flanker and flanker stages of training were not simply due to differences in stimulus eccentricity.

## Discussion

Preceding studies from the human psychophysics literature have examined the effects of various task manipulations on the ability of adult subjects to fine-tune their contrast discrimination faculties. Key avenues of exploration involved the introduction of a roving task paradigm (to increase levels of stimulus uncertainty), and the addition of flanker stimuli (to activate surrounding areas of the visual field, and embed the stimuli of interest within a fixed reference ‘context’). The effects of these task manipulations were found to vary considerably from one subject to the next, and tended to be crucially dependent on the specific task design- for example, the length and type of flankers presented [3], [14], and the duration of training [2].

In the current paper, we build upon the findings of Chen et al. [1], which previously demonstrated the ability of adult macaque monkeys to improve on a non-roving CD task. We identify additional parallels between the two species, in terms of the degree to which perceptual learning occurs, and the circumstances under which it develops. In cases where our task design differed slightly from those used in the human studies, the possible impact of these differences is addressed over the course of the discussion.

### Behavioural changes during the roving task

Our data show that under roving conditions, perceptual learning occurred for both monkeys, although the changes differed slightly between the two animals (e.g. improvements in the PSE occurred for different sample contrasts between the monkeys). We found that for a sample of 30% contrast, performance levels during a roving task remained comparable to those previously seen during training on a non-roving task (described by Chen et al. [1]). The selective improvements observed for the 40% and 20% sample conditions in monkeys 1 and 2, respectively, did not occur at the expense of performance with other sample contrast conditions, and were thus the product of genuine CD learning.

Adini et al. [3] neither observed improvements in performance when naïve observers were trained on the MBT roving task, nor saw improvements among subjects who had previously received training on a blocked task before embarking on the MBT task. In Yu et al.'s MBT roving task [2], naïve subjects delivered results that varied across individuals (as described in the introduction). The regimen followed by our subjects differed slightly from each of these groups of human subjects, as our monkeys were first exposed to a non-roving task, followed by a challenging version of the roving task (the MBT method). Moreover, to keep the task manageable for our monkeys, we used three sample contrasts, whereas Yu et al. [2] used four reference contrasts, and Adini et al. [3] used seven.

Nonetheless, on the whole, our observations matched those seen in human subjects by Yu et al. [2] and Adini et al. [3]: improvement was possible, albeit to a limited degree; it took place under only a subset of conditions; and results were not fully consistent between subjects.

### Addition of flanker stimuli

In either subject, when performance was assessed solely within the flanker training period, without regard for that seen during the preceding pre-flanker period, the proportion of correct trials and the slope of the psychometric function increased significantly across all sample contrasts for both subjects. Improvements were also observed in the slope and PSE for certain sample contrast conditions, which depended on the subject.

However, when the flanker period was assessed relative to the pre-flanker period, a striking divergence in the pattern of performance between the two subjects emerged. For monkey 1, flankers induced a brief worsening of performance, followed by a rapid return to pre-flanker levels, and a subsequent surge in performance above that seen in the absence of flankers. This matched the findings of a human psychophysics study by Adini et al. [13], which examined the effects of flanker training on CD thresholds, and found that while training with flankerless stimuli produced no significant improvement, the addition of flanker stimuli during training yielded reductions in threshold of ∼50%.

For monkey 2, the addition of flankers triggered a substantial decrease in performance which, throughout the course of flanker training, never completely recovered to pre-flanker levels. This result was closer to that reported by Yu et al. [2], in which flankers were unable to lower CD thresholds under either non-roving or roving conditions.

Tsodyks et al. [14] found that CD thresholds of Gabor stimuli could be modulated by the length of flanker chains. As the size of the flankers used in the main section of our experiment differed between subjects, this factor may partly explain the flanker-induced discrepancy in performance between our monkeys (a steep drop for monkey 2, despite a rapid gain for monkey 1). Our control experiment (using the same stimulus parameters in monkey 2 as those used for monkey 1) was intended to address this question; however, we cannot entirely rule out the possibility that the initial difference in stimulus parameters prompted monkey 2 to adopt different a task strategy from monkey 1, which persisted during the control task. For example, in theory, it is conceivable that while monkey 1 maintained a clear distinction between central and flanking stimuli, correctly basing his discriminations on comparisons of central stimuli, and using the flanker stimuli as visual aids, monkey 2 may instead have perceived the flankers as being part of the stimuli to be compared- hence the addition of flankers would effectively have increased unwanted noise in the signal. If so, then the strategy used by monkey 2 may have ‘carried over’ to the task with stimuli at 4.6° eccentricity, preventing him from discovering or developing the strategy that was successfully employed by monkey 1.

The use of a fixed flanker contrast raised the concern (voiced by Yu et al. [2]) that observers might not have carried out the task through a comparison of the absolute contrasts of sample and test stimuli, but rather, by taking note of the difference in contrast between the *flankers* and the central stimuli during each stimulus presentation interval, and then comparing the *size of the differences* between intervals. If so, then subjects may have built up ‘difference templates’ over the course of training. Yu et al. addressed this possibility by carrying out two versions of the task- one in which flanker contrasts were ‘jittered’ randomly from trial to trial, but remained the same during both stimulus presentation intervals per trial; and one in which the flanker contrast was fixed at 40%. After analysing their data, they felt that this precaution had been unnecessary as the two versions of the task yielded indistinguishable results. Being unable to explicitly instruct our monkeys to make their comparisons between the central stimuli, rather than between flanker stimuli, we did not implement the ‘jittered flanker’ paradigm, and thus cannot conclusively rule out the possibility that our monkeys based their decisions on a comparison of contrast differences. Given that non-jittered and jittered flanker approaches have yielded similar results in humans [2], and judging by the overall similarity of our results to those found in humans, we would predict that practice with jittered flanker contrasts would produce results that are comparable to what we report in this paper using a fixed flanker contrast.

### Removal of flankers

Changes in performance during training on the flanker task- whether in the form of improvements or deteriorations- did not persist in the absence of flankers. In monkey 1, performance on the flankerless task was even slightly worse after a period of flanker training. This result closely mirrors that reported by Yu et al. [2], in which practice with flankers resulted in increases in contrast thresholds and partial reversals of pre-flanker improvements in performance.

### Differences from human studies

The current experiment differed in several respects from those used in the human experiments. In human studies, subjects were made explicitly aware of the task requirements; our monkeys, on the other hand, received instruction through a prolonged process of conditioning, trial and error, and reward association. Hence, improvements that occurred during the early stages of training were likely to have resulted from a combination of general task learning (learning to pay attention to the sample contrast) and fine perceptual learning (making subtle contrast discriminations). Under certain circumstances, it is possible to dissociate the two forms of learning from one another, as was done by Chen et al. (2013) [1]: improvement for the easiest test contrast conditions was used as a proxy for non-specific task learning, and any improvements that exceeded this ‘base level’ of learning were attributed to perceptual learning ‘proper.’ In reality, improvements in both types of learning are likely to co-occur and may proceed at different speeds, depending on the monkeys' focus of attention and task strategy. For example, it has been suggested that human subjects might dedicate themselves to learning one subset of task conditions at a time, before expanding their focus to another subset [3]. Implementation of the roving task required the introduction of conditions that involved fine contrast discriminations, rather than coarse ones, precluding the derivation of a proxy measure of general roving task learning with which to resolve the two distinct components of learning.

Human psychophysical studies typically use a two-alternative-forced-choice (2AFC) staircase procedure to determine threshold levels of contrast discrimination [2], [3], [14], [20], in which contrast levels of the stimuli are continually adjusted to match the subjects' behavioural performance. Such a paradigm is feasible in human subjects, who can comprehend the task requirements from the outset; it would have been more difficult for our monkeys to master the task when presented with such rapidly changing stimuli. Hence, to ensure that our subjects learnt the task, the contrast of the test stimulus was drawn from a restricted subset of twelve values per sample contrast. This yielded psychometric functions, from which comparable measures of performance (such as the slope, the PSE, and the upper and lower thresholds) were derived. These differences in task design notwithstanding, our results closely corroborate those from the human literature, and constitute a valuable addition to future meta-analyses of perceptual learning of contrast discrimination.

### Neuronal mechanisms of perceptual learning

Several candidate theories for the mechanisms underlying PL have been put forward; among them is the ‘reverse hierarchy theory of learning’ (RHT) by Ahissar and Hochstein [4]. The RHT posits that top-down mechanisms such as attention are responsible for selective alterations of appropriate neuronal populations. With practice, changes propagate from higher- to lower-level neuronal populations in the visual hierarchy. Gradually, areas that are responsible for making relatively fine perceptual distinctions become ‘wired up’ more efficiently. Kuai et al. [9] and Zhang et al. [8] suggested that the RHT model might be compatible with their findings, as the regular temporal ordering of reference contrasts might facilitate the ‘tagging’ of stimuli and enable top-down attentional mechanisms to target low-level cortical regions during PL-induced plasticity. Adini et al. [3] noted that improvements may reflect changes in the shape of the contrast transducer function of individual neurons; alternatively, they may result from changes in connectivity *between* neurons, through an optimisation in the selection and gating of subpopulations of channels.

In the current study, if training with flankers had engaged exactly the same cognitive processes as those used in the absence of flankers, then one should not expect to see a reversal in performance after their removal. Based on our observations, the neuronal mechanisms used to perform the task in the absence of flankers appeared to be distinct from those used in the presence of flankers. Centre-surround modulations of activity in low-level cortical areas such as V1 may have intensified during the flanker task, creating local changes in the balance of excitatory and inhibitory horizontal inputs to V1 neurons [21] and giving rise to differences in performance between flankerless and flanker training periods. It is also possible that the presence of flankers temporarily altered the connectivity between low-level and intermediate areas such as V4, causing the ‘readout’ of distinct subpopulations of neurons from low-level regions. Such a change in readout could be achieved through modulations of oscillatory activity that are induced by surround stimulation [22], which could then alter coherence-based communication between neuronal pools [23]. Finally, computational mechanisms within cortical regions such as V4 may have allowed the pooling and processing of incoming information to vary [24], according to the demands of the task.

In summary, we found that perceptual learning of contrast discrimination is possible under roving conditions in macaque monkeys; furthermore, the addition of flanker stimuli does not result in permanent improvements in CD that are uniform across subjects, but instead triggers temporary changes, the effects of which differ between individuals. The findings presented here serve to broaden our understanding of inter-species similarities in visual perception, and pave the way for future explorations of PL at the neuronal level in the adult primate.

## Supporting Information

Figure S1 *P_reporthigher_* for ‘response conflict conditions,’ plotted against session number. These conditions necessitated different responses, depending on the sample contrast. A: monkey 1; B: monkey 2. Within each subplot, the leftmost data points indicate subjects' performance during the non-roving task, while those to the right indicate performance during the roving task. Black markers: conditions in which a 30% contrast sample was presented; grey markers: conditions with a 20% or 40% sample. Note that the 38% test contrast condition was only introduced at the start of roving training, and thus no data were available for this test contrast during the non-roving period. A visual comparison revealed that at the beginning of training on the roving task, subjects' responses to a given test contrast tended to be similar, regardless of the actual contrast of the sample, i.e. responses appeared to have been based on the 30% reference that was used during the non-roving task. A divergence in data points between response conflict conditions (represented by differences in slope between fitted lines within individual subplots) indicated that learning occurred under roving conditions i.e. the monkeys learnt to correctly make their comparison based on the sample contrast. Additionally, the data points appeared to diverge more between the sample conditions for monkey 2 than for monkey 1, indicating that learning was slightly more pronounced for monkey 2.
(EPS)

Figure S2 *P_reporthigher_* plotted against session number, for each test contrast condition (coded by colour). A & B: 20% sample contrast; C & D: 30% sample contrast; E & F: 40% sample contrast. Training was initially carried out without flankers, then a period of flanker training commenced, followed by several sessions without flankers. Left column: monkey 1; right column: monkey 2. Lines represent the running average across three consecutive sessions.
(EPS)
